# A survey of surgical team members’ awareness and perceptions toward the implementation of the surgical safety checklist in gynecological and obstetrical operations

**DOI:** 10.1097/MD.0000000000026731

**Published:** 2021-07-30

**Authors:** Junming Gong, Bo Sheng, Ce Bian, Lingyun Yang

**Affiliations:** a Key Laboratory of Birth Defects and Related Diseases of Women and Children (Sichuan University), Ministry of Education; b Department of Central Operating Unit, West China Second University Hospital; c West China School of Nursing; d Department of Anesthesiology, West China Second University Hospital; e Department of Gynecology and Obstetrics, West China Second University Hospital, Sichuan University, Chengdu, Sichuan, China.

**Keywords:** attitudes, gynecological and obstetrical operation, perceptions, surgical safety checklist, surgical team members

## Abstract

The World Health Organization Surgical Safety Checklist was developed to improve communication in perioperative care, reduce mortality and complications of patients, and ensure the consistent use of procedures for safe surgery. Despite the increased awareness of the checklist, the implementation compliance is reported as low and the degree of completeness varies. This study aimed to explore the possible supportive factors for the effective implementation and to identify potential awareness and barriers to its implementation in gynecological and obstetrical operation.

A survey using a cross-sectional design that included surgeons, anesthetists, and operating room nurses was performed. We used an online link to distribute the survey to all eligible surgical team members in our hospital. The survey contained various aspects of perceptions on the Surgical Safety Checklist and an open-ended question that allowed respondents to offer their opinions on the topic.

The overall self-reported awareness of the checklist within each professional group was high. The awareness of surgeons was lower than that of operating room nurses, particularly in the Time-out section. Most participants believed that operating room nurses ranked the highest compliance to the protocols, while surgeons stayed the lowest. Active leadership with experienced operating room nurses, good training for surgical team members, and simplification of the checklist would be the positive factors for the effective implementation.

Although there is a high acceptance and adequate self-reported awareness of the Surgical Safety Checklist, it is not always possible to implement it successfully. Our findings suggest that with experienced and effective leadership, barriers to implementation can be overcome. With positive perception and commitment, the Surgical Safety Checklist is easy to implement and it can make a profound improvement on the safety of surgical care. Moreover, a strategy of repetitive training and assessment on the part of the involved health care professionals may be necessary to further improve patients’ safety during surgery.

## 1 Introduction

Surgery is an important treatment option for many gynecological and obstetrical diseases; however, medical errors which occur during the surgery are the main reasons for serious complication developed due to surgery.^[1]^ It is reported that medical errors and adverse events in surgical patients are estimated to be highly preventable in 48% of the cases.^[2]^ One of the key elements in the surgical risk management approach to a safer hospital environment is the use of a surgical safety checklist. The World Health Organization (WHO) Surgical Safety Checklist (SSC) was developed to improve communication in perioperative care, to reduce mortality and complications of patients, and to ensure the consistent use of procedures for safe surgery.^[3]^

The WHO SSC is administrated in three sections: “Sign-in,” “Time-out,” and “Sign-out.” “Sign-in” includes verification of the patient's identity, the surgical procedure and site, and consent before induction of anesthesia. Besides, the known allergy and risk of blood loss are reviewed. “Time-out” includes further confirmation of correct operation on the correct patient and site before the skin incision. There is a review of anticipated critical events, antibiotic prophylaxis, equipment availability, and essential imaging results. “Sign-out” includes the record of the procedure, the completion of the instrument count, and the labeling of specimens before the patient leaves the operating room (OR). Any issues with equipment and postoperative prescriptions and instructions are reviewed. There is increasing evidence that a positive influence on patients’ outcomes is more likely if SSC items are optimized.^[4–6]^ Improved communication and shared responsibility within the health care teams may contribute to reducing medical error and adverse events.^[7,8]^ The WHO SSC procedures of 3 sections have been packaged and implemented over 10 years in our hospital to reduce medical errors and improve the prognosis of all patients.

Despite the increased awareness of the SSC, the implementation compliance is reported as low and the degree of completeness varies. It is reported that the possible reason for this could be a lack of positive role models or less than enthusiastic team members, hierarchical barriers, limited knowledge of correct usage, and inappropriate implementation procedures.^[9]^ In the Obgyn operation room unit, there are many emergency surgeries, such as salpingectomy or salpingostomy for ectopic pregnancy and cesarean section for fetal distress, umbilical cord prolapse, placental abruption, etc. Clinical mistakes and errors are more likely to occur in emergency cases because the SSC protocols were not accurately implemented. Moreover, the surgeon acts as the leadership of the surgical team, and the awareness and perceptions of the surgeon are particularly important. It is reported the compliance towards safety protocols of surgeon stays at the lowest level in the surgical team.^[10–12]^ Thereby, active involvement in the implementation phase, as well as continuous training, is presumed to greatly impact the compliance and acceptance by all health care team.^[13,14]^

In this study, we investigated the awareness and perceptions of the implementation of the SSC by frontline medical professionals of the department of gynecology and obstetrics in our hospital. This study aimed to explore the possible supportive factors for the effective implementation of the SSC and to identify potential awareness and barriers to its implementation.

## 2 Methods

All ethical approval and consent procedures were approved by the Medical Ethical Committee of West China Second University Hospital, Sichuan University (Ethical approval No. 2020–140). We confirmed that all research was performed following relevant regulations and informed consent was obtained from all participants.

We surveyed surgical team members’ awareness and perceptions using a cross-sectional design that included surgeons, anesthetists, and OR nurses in Western China Second University Hospital. All participants worked in the central operating unit. This survey had to do with the principle of anonymity, and authors did not have access to information that could identify individual participants during or after data collection. The attitude scales of this survey referred to a previous article about the implementation status of the SSC.^[15]^

The survey contained various aspects of perceptions on the SSC and an open-ended question that allowed respondents to offer their opinions on the topic. The items are listed in Table 1. The survey also included questions about participants’ characteristics such as profession, experience, and gender. The survey was pilot tested by 10 research fellows and medical staff who reviewed the functionality of the adequacy and relevance of the questions (Table 2). We started to use an online link to distribute the survey to all eligible surgical team members in our hospital in March 2020. The deadline to submit the questionnaire is the end of August 2020.

**Table 1:** Questionnaire of SSC compliance.

Awareness of the SSC	Are you aware of the implementation of the SSC? (Score 0–10)
	Are you aware of the implementation in the Sign-in section? (Score 0–10)
	Are you aware of the implementation in the Time-out section? (Score 0–10)
	Are you aware of the implementation in the Sign-out section? (Score 0–10)
Compliance with the SSC	
	Who do you think ranks the highest compliance to the SSC? (surgeons, anesthetists, OR nurses)
	Who do you think ranks the lowest compliance to the SSC? (surgeon, nurses, anesthetists, OR nurses)
Leader for the SSC	Who should be the leader to implement the Sign-in protocol?
	Who should be the leader to implement the Time-out protocol?
	Who should be the leader to implement the Sign-out protocol?
Subjective attitude to SSC	Is it useful to apply the SSC in OR?
	What is your opinion about the positive aspects to apply the SSC?
	What is your opinion about the factors that influence SSC compliance?

**Table 2:** Pilot test of the functionality of the attitude scales of this survey.

	Adequacy	Relevance	Practicability	Simplicity
No.1	Yes	Yes	Yes	Yes
No.2	Yes	Yes	Yes	Yes
No.3	Yes	Yes	Yes	Yes
No.4	Yes	Yes	Yes	Yes
No.5	No	Yes	Yes	Yes
No.6	Yes	Yes	Yes	Yes
No.7	Yes	Yes	Yes	Yes
No.8	Yes	Yes	No	Yes
No.9	Yes	Yes	Yes	Yes
No.10	Yes	Yes	Yes	Yes

Statistical analysis was performed using SPSS software. Pearson Chi-square or Fisher exact test was applied to compare the difference in proportions. *P* < .05 was statistically significant.

## 3 Results

In the central operating unit, 267 staff were invited to participate in the survey. The general characteristics of all respondents are summarized in Table 3. Answers were received from 85 surgeons, 86 anesthetists, and 96 OR nurses. The participants consisted of 218 (81.65%) women and 49 (18.35%) men. The median age was 30 years, and 30.71% of staff had been working in the OR center for over 10 years.

**Table 3:** General demographics of respondents.

	Total (N = 267)	Surgeon (N = 85)	Anesthetists (N = 86)	OR nurses (N = 96)
Age (median, range)	30 (22–59)	33 (22–54)	31 (21–54)	29 (23–59)
Gender (%)				
Males	49 (18.35)	25 (29.41)	18 (20.93)	6 (6.25)
Females	218 (81.65)	60 (70.59)	68 (79.07)	90 (93.75)
Profession				
Resident/Trainee	145 (54.31)	29 (34.12)	49 (56.98)	67 (69.79)
Attending/Consultant	85 (31.84)	36 (42.35)	21 (24.42)	28 (29.17)
Associate professor/Professor	37 (13.85)	20 (23.53)	16 (18.60)	1 (1.04)
Professional experience (%)				
0–5 yr	127 (47.57)	36 (42.35)	45 (52.33)	46 (47.92)
6–10 yr	58 (21.72)	20 (23.53)	17 (19.77)	21 (21.88)
11–20 yr	58 (21.72)	20 (23.53)	16 (18.60)	22 (22.92)
Over 20 yr	24 (8.99)	9 (10.59)	8 (9.3)	7 (7.28)

**Figure 1:**
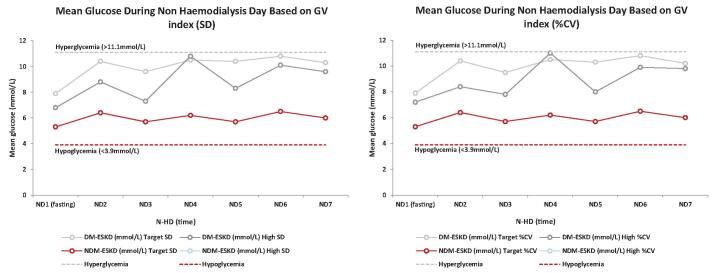
Self-reported awareness of the implementation of the SSC. The awareness of surgeons is lower than that of OR nurses in the Time-out section (Score of 6.71 vs 8.07, ^∗^*P* < .001). The awareness of implementing the SSC in the Sign-in section (Score of 9.58) is significantly higher than that in the Time-out (Score of 7.45) and Sign-out (Score of 7.51) sections (^†^*P* < .001).

**Figure 2:**
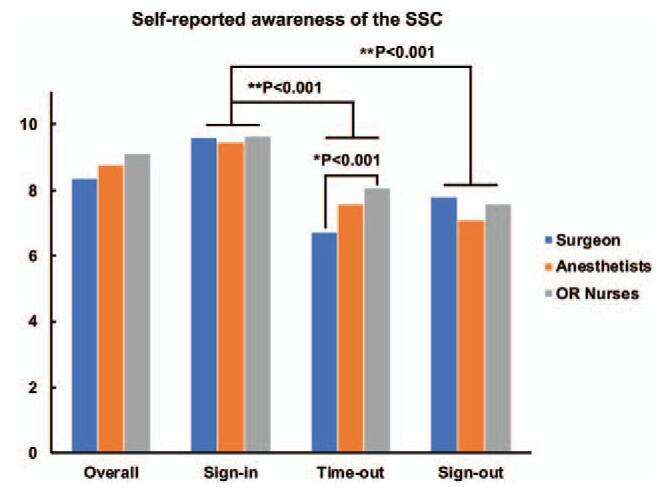
The compliance to the SSC in each professional. OR nurses ranked the highest compliance to the SSC protocols (^∗^*P* < .001). Surgeons stayed the lowest compliance to the SSC protocols (^†^*P* < .001).

The overall self-reported awareness of the SSC within each professional group was high with an average score of 8.76. However, the awareness of surgeons was lower than that of OR nurses, particularly in the Time-out section (Score of 6.71 vs 8.07, *P* < .001). Most of the respondents believed that they had an active perception in implementing the SSC in the Sign-in section (Score of 9.58) rather than that in the Time-out (Score of 7.45) and Sign-out (Score of 7.51) sections (*P* < .001) (Fig. 1 and Table 4). Furthermore, as many as 82.4% (220/267) participants believed that OR nurses ranked the highest compliance to the SSC protocols, while 77.9% (208/267) participants agreed that surgeons stayed the lowest compliance to the SSC protocols (Fig. 2 and Table 5).

**Table 4:** Self-reported awareness (average scores) of the implementation of the SSC.

	Overall	Sign-in	Time-out	Sign-out
Surgeon	8.37 ± 0.17	9.60 ± 0.11	6.71 ± 0.25	7.80 ± 0.25
Anesthetists	8.78 ± 0.15	9.49 ± 0.13	7.60 ± 0.24	8.07 ± 0.28
OR nurses	9.14 ± 0.20	9.65 ± 0.15	8.07 ± 0.28	7.60 ± 0.34

**Table 5:** The compliance to the SSC in each professional from the survey.

	High compliance	Low compliance
Surgeon	19 (7.1)	208 (77.9)
Anesthetists	28 (10.5)	35 (13.1)
OR nurses	220 (82.4)	24 (9.0)

**Figure 3:**
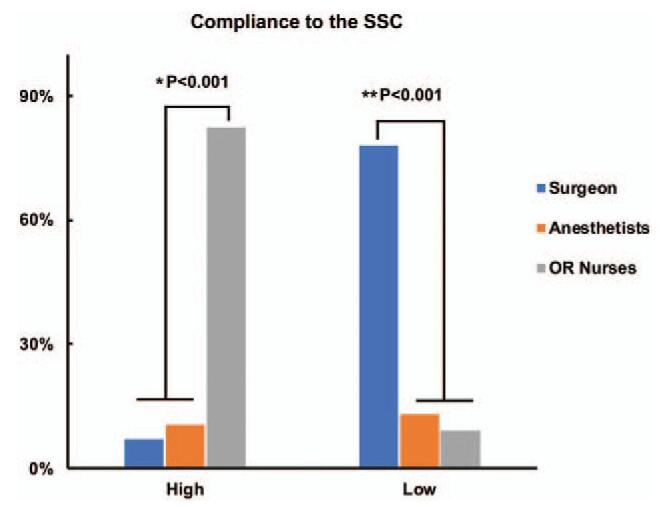
The leader to implement the SSC in each section. 55.1% (147/267) of participants believed that anesthetists should be the leader to implement the Sign-in protocol. 40.8% (109/267) and 37.1% (99/267) were certain about both surgeon and OR nurses as the leader in the Time-out protocol. 62.5% (167/267) agreed that OR nurses should be the leader in the Sign-out protocol.

In total, 55.1% (147/267) of participants believed that anesthetists should be the leader to implement the Sign-in protocol, while 62.5% (167/267) agreed that OR nurses should be the leader in the Sign-out protocol. Interestingly, 40.8% (109/267) and 37.1% (99/267) were certain about both surgeons and OR nurses as the leader in the Time-out protocol (Fig. 3 and Table 6).

**Table 6:** The leader to implement the SSC in each section from the survey.

	Sign-in	Time-out	Sign-out
Surgeon	57 (21.3)	109 (40.8)	35 (13.1)
Anesthetists	147 (55.1)	59 (22.1)	65 (24.3)
OR nurses	63 (23.6)	99 (37.1)	167 (62.5)

85% (227/267) of respondents believed that they would be more likely to comply with the use of the SSC, whereas 15% (40/267) of respondents thought that the SSC caused delays, and 11% (30/267) did not believe it worked. 242 respondents answered the open-ended questions and included their reflections on awareness and perceptions of the SSC protocols and safety issues. Most participants noted that the endorsed implementation of the SSC prevented mistakes in the operation room, such as wrong patient's identity, the uncertainty of correct site, wrong position of the patient, and medication allergy. Besides, some positive and negative factors that may influence the use of the SSC were described (Table 7). Many participants felt that they would be more likely to comply with the use of the SSC if the surgical team had an experienced OR nurse who would like to be the organizer in using the SSC. Moreover, most respondents suggested shortening the SSC procedure duration might increase compliance. We also identified the negative factors and barriers to the implementation of SSC. First, we found that some surgical team members lacked effective training about the implementation of the SSC, so they did not seriously realize its importance. Second, on the occasion of an emergency, the SSC protocols were not accurately implemented, and most nurses complained that surgeons were always in a hurry and did not want to reply to the checklist questions, particularly in the Time-out section. Interestingly, most surgeons supported that it was a waste of time to stop and answer the checklist questions in the Time-out section, even if they have done it previously. Lastly, some participants described that the content of SSC was strange and complicated, and it took so long to finish.

**Table 7:** Main factors influencing the implementation of SSC.

Positive factors	Negative factors
Active leadership: experienced OR nurse	Lack of good training
	Lack of time: emergency, surgeons in a hurry
	Strange and complex content of the SSC

## 4 Discussion

Each of the surgical teams in gynecological and obstetrical OR routinely implemented the SSC into their daily procedures. Awareness of the SSC is integral in its successful adoption.^[16]^ The overall awareness of the implementation of the SSC within each professional group was high with an average score of 8.76 (range from 0 to 10), which parallels those of previous studies that showed high levels of awareness of the SSC usage.^[17,18]^ Most of the respondents were satisfied with the use of the SSC, highlighting the contribution in minimizing surgical errors. This result was found to be following respect to the rate of SSC used and the subjective belief of healthcare professionals. Furthermore, we found that OR nurses showed the highest levels of awareness rather than surgeons, because a large initiator of implementation of the SSC was introduced via OR nurses, who were less autonomous in their scope of practice and were mandated by their clinical managers to use the SSC.

On the contrary, participants in the current survey noticed that surgeons’ level of awareness was at the bottom, particularly in the phase of Time-out. We found that perceptions towards Time-out protocols were at the lowest level, and 77.9% of participants agreed that surgeons stayed the lowest compliance to the SSC protocols in our hospital. The SSC of Time-out phase was not completed or was left incomplete, because some surgeons did not comprehend the importance of the SSC adoption, and they thought that the checklists have to do with OR nurses’ duties only, therefore they do not need to participate in the completion. Whereas surgeons always complained that the Time-out protocols caused delays in the operation especially on emergency occasions, such as ectopic pregnancy, ovarian cyst torsion, placental abruption, placenta previa with massive hemorrhage, or fetal distress, etc. Previous studies showed that nurses’ and surgeons’ acceptance of the SSC could reveal insufficient equipment standardization and improve cooperation.^[19,20]^

There is a recognition that an experienced leader in each section of the SSC implementation is necessary, and the leader in the section of Sign-in, Time-out, and Sign-out should be the anesthetist, surgeon, and OR nurse respectively.^[21]^ In this survey, participants believed that both surgeons and OR nurses were the leader in the Time-out section. Even some surgeons waited for the OR nurses to initiate and complete the Time-out protocols before skin incision. Although the growing evidence supporting the SSC use, this study suggested that there were mixed attitudes regarding its utility from frontline medical professionals. 11% of respondents, particularly surgeons and anesthetists, believed that the SSC is unlikely to improve surgical safety in their patient population because they felt the SSC had the potential to distract them from their clinical duties and they were somewhat burdened by the task of completing the SSC. Even when OR staff complied with the components of the SSC, other team members were sometimes obstructive, inattentive, and preoccupied with other tasks, which may reflect a lack of belief in the utility of the SSC. In addition, we noticed that the motivation for completing the SSC appeared to differ from OR nurses and other staff. OR nurses emphasized that the SSC was a mandated protocol and, thus, needed to be completed. Surgeons and anesthetists were more concerned with their clinical tasks. That is why OR nurses always forced surgeons and anesthetists to complete the SSC even in the Sign-in and Time-out section.

The present study highlighted various factors influencing compliance with the use of the SSC. It was reported that the surgeons and anesthetists were the members of the surgical team who did not fully comply with the SSC implementation or who were the most reluctant to change their behaviors.^[22]^ However, we noticed that if there was an experienced OR nurse who had the primary responsibility for initiating the use of each SSC section, other members tended to be more obedient to the procedures. It is recognized that experienced OR nurses comprehend the cooperation within the whole surgical team is essential. The importance of communication and teamwork had been demonstrated as an integral part of health care provision.^[23]^ Consequently, active leadership is important for the successful application and sustainability of a checklist as well as for regular inspections and feedback.

It should be noted that the majority of participants reported that the lack of time and training is the most important barrier to complete the SSC. The introduction of the SSC implementation evoked criticism because some surgeons found that the SSC procedure often interfered with the workflow of existing routines and delayed their operation time. They considered that it was a waste of time to answer the questions in the Time-out section before they made the first incision, especially on emergency occasions. Therefore, the subjective compliance of some senior surgeons stayed at a low level, and they were resistant to change their habits when implementing the SSC. Surgeons’ commitment is particularly important to successful SSC implementation,^[24]^ which suggested that surgeons need to be both supported and trained to elevate the level of compliance. It is reported that surgeons’ level of compliance was strongly related to training on the SSC, and education and training during SSC implementation enabled the surgeons to comprehend the importance of the SSC adoption.^[25]^ In this survey, 1 participant reported that surgeons ignored the Time-out section of the checklist and forgot to perform fallopian tube ligation, which was requested by a patient with pernicious placenta previa during the cesarean surgery. Another adverse event was recorded that gauze was left in the patients’ vagina after laparoscopic hysterectomy causing infection of the vaginal margin because the surgeon and OR nurse did not complete the instrument count in the Sign-out section. The awareness and positive attitudes towards the purpose and importance of the SSC suitably put surgical members in OR. Besides, the attitudes of surgical team members toward the SSC influenced the willingness to participate in training. To achieve better compliance, training should become an integral part of each surgical team and be performed continuously. Therefore, to further facilitate the use of the SSC, we aim to produce a video with proponents. This video is to become an integral part of effective training and to demonstrate good practice, study data, and case studies where the SSC enhanced patient safety. Furthermore, an additional factor reported as hurting the compliance of the SSC adoption was its strange and complex content. It is suggested that the content of the SSC needs to be supported by scientific evidence and to be written clearly, using understandable words preferably embedded within existing processes.^[26]^ Some participants in this survey suggested that the SSC requires simplification in emergency cases to allocate enough time for the operation. Therefore, it will affect the compliance of each professional.

A limitation of the current study was the fact that there was no baseline survey before the implementation of the SSC and it was only conducted over a brief period. We assessed compliance in terms of SSC use; however, we did not investigate the quality of SSC use. The attitudes of health care providers may differ substantially owing to differing baseline perioperative procedures before implementation of the SSC. Therefore, the findings cannot be generalized because this study is susceptible to a professional desirability bias.

In conclusion, this study provides information about the current awareness and perceptions towards the implementation of the SSC in gynecological and obstetrical operations. The implementation and sustainability of SSC remain a major challenge. Although there is a high acceptance and adequate self-reported awareness of the SSC, it is not always possible to implement it successfully. Our findings suggest that with experienced and effective leadership, barriers to implementation can be overcome. With positive perception and commitment, the SSC is easy to implement and it can make a profound improvement on the safety of surgical care. Moreover, a strategy of repetitive training and assessment on the part of the involved health care professionals may be necessary to further improve patients’ safety during surgery.

## Acknowledgments

We are sincerely grateful for the clinical and statistical assistance provided by Dr Xiaorong Qi, Dr Xi Tan, Dr Han Huang, and Nurse Li Wan from West China Second University Hospital.

## Author contributions

**Conceptualization:** Ce Bian, Lingyun Yang.

**Data curation:** Junming Gong.

**Formal analysis:** Junming Gong, Bo Sheng, Lingyun Yang.

**Funding acquisition:** Ce Bian.

**Investigation:** Junming Gong, Bo Sheng.

**Methodology:** Ce Bian, Lingyun Yang.

**Writing – original draft:** Junming Gong, Lingyun Yang.

**Writing – review & editing:** Lingyun Yang.
